# Typology and Circuitry of Suppressed-by-Contrast Retinal Ganglion Cells

**DOI:** 10.3389/fncel.2018.00269

**Published:** 2018-08-27

**Authors:** Jason Jacoby, Gregory William Schwartz

**Affiliations:** ^1^Department of Ophthalmology, Feinberg School of Medicine, Northwestern University, Chicago, IL, United States; ^2^Department of Physiology, Feinberg School of Medicine, Northwestern University, Chicago, IL, United States; ^3^Department of Neurobiology, Weinberg College of Arts and Sciences, Northwestern University, Chicago, IL, United States

**Keywords:** retina, retinal ganglion cells, typology, suppressed-by-contrast, contrast suppression, feature selectivity, encoding visual information

## Abstract

Retinal ganglion cells (RGCs) relay ~40 parallel and independent streams of visual information, each encoding a specific feature of a visual scene, to the brain for further processing. The polarity of a visual neuron’s response to a change in contrast is generally the first characteristic used for functional classification: ON cells increase their spike rate to positive contrast; OFF cells increase their spike rate for negative contrast; ON-OFF cells increase their spike rate for both contrast polarities. Suppressed-by-Contrast (SbC) neurons represent a less well-known fourth category; they decrease firing below a baseline rate for both positive and negative contrasts. SbC RGCs were discovered over 50 years ago, and SbC visual neurons have now been found in the thalamus and primary visual cortex of several mammalian species, including primates. Recent discoveries of SbC RGCs in mice have provided new opportunities for tracing upstream circuits in the retina responsible for the SbC computation and downstream targets in the brain where this information is used. We review and clarify recent work on the circuit mechanism of the SbC computation in these RGCs. Studies of mechanism rely on precisely defined cell types, and we argue that, like ON, OFF, and ON-OFF RGCs, SbC RGCs consist of more than one type. A new appreciation of the diversity of SbC RGCs will help guide future work on their targets in the brain and their roles in visual perception and behavior.

## Introduction

Suppressed-by-Contrast (SbC) Retinal ganglion cells (RGCs) were first discovered in 1967 in the cat (Rodieck, 1967) and rabbit retina (Levick, 1967). Since that time, SbC RGCs have been recorded and further characterized by other researchers in the retina of the cat (Mastronarde, 1985; Troy et al., 1989), rabbit (Sivyer et al., 2010, 2011), macaque (de Monasterio, 1978). SbC responses have also been found in visual neurons in the brain, including in the dorsal lateral geniculate nucleus (dLGN) of the macaque (Tailby et al., 2007) as well as both dLGN (Piscopo et al., 2013) and primary visual cortex (Niell and Stryker, 2008) of the mouse. Previous research efforts have postulated that SbC visual neurons in the retina and higher visual centers may play a role in accommodation, contrast gain modulation and saccadic suppression (Rodieck, 1967; Troy et al., 1989; Tien et al., 2015).

SbC RGCs were only recently identified in the mouse retina; in 2015 two research groups presented an RGC that exhibited contrast suppression profiles, and both groups claimed to have found the SbC RGC in the mouse retina (Jacoby et al., 2015; Tien et al., 2015). While both SbC RGCs do share similar properties, a direct comparison reveals distinct differences in morphology, function, synaptic inputs and circuit connectivity.

## Typology of Sbc RGCs

Identification of neuronal cell types is critical to understanding the brain because they specify a parts list from which circuits are assembled, and one important goal of current brain-mapping initiatives is to map neural connectivity by placing identified cell types into functional circuits (Sanes and Masland, 2015). The retina is a particularly powerful model system for cell typology and circuit mapping because of a rich history of work on typology across several mammalian species. Genetic tools have accelerated the pace of discovery of new retinal cell types and their connections, particularly in the mouse.

With this fast rate of discovery comes a danger that some of the distinctions between similar cell types could be missed. Our principle argument is that two recent articles claiming to have identified the SbC RGC in mouse in fact identified different SbC RGCs. Like ON, OFF, and ON-OFF, SbC is a response polarity class comprising multiple distinct RGC types. At least two different SbC RGCs exist in the mouse retina, and we will review their similarities and differences from the perspectives of genetics, morphology and function. We will argue that one of this types, which we call the transient SbC, is identical to a RGC type that was recently discovered and named the ON delayed RGC (Mani and Schwartz, 2017).

## Targeting Sbc RGCs

Transgenic mice have become useful tools for targeting neuronal cell types throughout the central nervous system, but unfortunately, few of the transgenic lines currently available label single cell types (Martersteck et al., 2017). In the preliminary screening of a line that fluorescently labeled three different ganglion cell types (*CCK-cre*), Zhu et al. (2014) observed that one type shared morphological characteristics with an SbC RGC type in rabbit retina, known as the Uniformity Detector RGC. Tien et al. (2015) performed physiological recordings from fluorescently-labeled RGCs in the CCK-cre transgenic line using 2-photon laser-guided targeting, and they confirmed that a subset of labeled cells indeed exhibited a SbC response profile (Tien et al., 2015).

While the CCK-cre is the only transgenic line that contains fluorescently-labeled SbC RGCs that could be directly targeted, other studies non-genetically targeted SbC RGCs strictly by their physiology and morphology in transgenic lines, where SbC RGCs were not fluorescently labeled, but various amacrine cells (ACs) were labeled. In a different study performed concurrently to that of Jacoby et al. (2015) and Tien et al. (2015) physiologically targeted (by stereotyped light response profile and a sustained contrast suppression profile) SbC RGCs in both wild type or the CRH-cre transgenic retina where only CRH-positive ACs were genetically labeled. In two follow-up studies performed in 2016, both Lee et al. (2016) and Tien et al. (2016) targeted SbC RGCs based on their contrast-suppression and/or dendritic morphology in a different transgenic mouse line (*VGluT3-cre*) where an associated AC was labeled but SbC RGCs were not.

## Morphology

Morphology is a critical component in identifying distinct types of RGCs. This includes characteristics such as size of the dendritic area, diameter of the soma, stratification profile in the inner plexiform layer (IPL) and branch density (Bae et al., 2018). When SbC (Uniformity Detector) RGCs were described in the rabbit retina, one key morphological characteristic was the recursive nature of the cell’s dendritic arbor; many of the distal dendrites in the OFF arbor did not terminate there, but instead dove recurrently back to the ON arbor (Sivyer et al., 2010). The presence of recursive dendrites was used to argue that SbC RGCs in mouse are homologous to the rabbit Uniformity Detector RGC (Zhu et al., 2014; Tien et al., 2015). Mani and Schwartz (2017) identified a RGC type they called the ON delayed RGC, and they reported that these RGCs have a greater number and total length of recursive dendrites than the SbC RGC of (Jacoby et al. (2015); their Supplementary Figure S1; Mani and Schwartz, 2017). The full electron-microscopic (EM) reconstruction of all RGCs in a block of mouse retina (Bae et al., 2018) provides a valuable reference for comparing the morphologies of RGCs originating from different publications to determine if they belong to the same or different types. We compared images from our own lab, and an image stack and data graciously provided Nai-Wei Tien and Daniel Kerschensteiner, to the online EM database, focusing on the details of the stratification pattern in the IPL.

**Figure 1:**
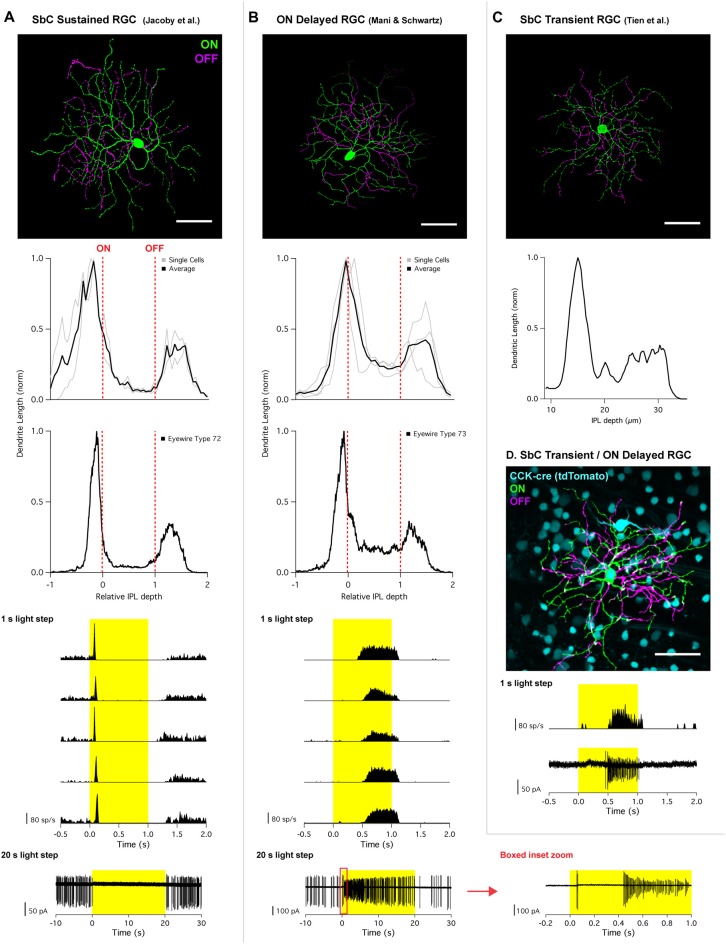
Morphology and physiology of Suppressed-by-Contrast (SbC) retinal neurons. **(A)** SbC sustained retinal ganglion cell (RGC) identified by Jacoby et al. (2015) and **(B)** ON delayed RGC identified by Mani and Schwartz (2017). Top image; representative RGC image showing ON dendrites (green) and OFF dendrites (magenta). Middle section; stratification profiles of several individual cells (gray) overlayed with an average trace (black), followed by stratification profile of corresponding Eyewire cell types. For all stratification profiles, the vertical red dotted lines represent the ON and OFF choline acetyl transferase (ChAT) bands. Bottom traces; peristimulus time histogram (PSTH) of 1 s light step from darkness from five different cells from five different retinas. Bottom traces shows response to 20 s light step in current clamp configuration (SbC sustained RGC) and cell attached mode (ON delayed RGC). For 20 s light step for the ON delayed RGC, a zoomed in trace of the red box inset is plotted to the right. **(C)** SbC transient RGC identified by Tien et al. (2015). Top image; representative image of an SbC transient RGC (ON dendrites = green; OFF dendrites = magenta). Middle section; stratification profile of representative image above using z-axis fluorescent profile. **(D)** ON delayed/SbC transient RGC recorded in CCK transgenic mouse line. Top image; representative image with CCK labeling with tdTomato (cyan), ON dendrites (green), OFF dendrites (magenta). Bottom traces; PSTH and cell attached spikes derived from 1 s light step from darkness recorded from the cell depicted above. All scale bars = 50 μm. Permission from the copyright holders was obtained for use and modification of previously published figures.

The dendrites of the SbC RGC that was identified by Jacoby et al. (2015) stratified proximal to the ON choline acetyl transferase (ChAT) band and distal to the OFF ChAT band (Figure 1A). Like other bistratified RGCs, the most of the dendritic length was confined to the ON and OFF strata with 16.4 ± 6.8% between strata for two reconstructed cells. This stratification profile is consistent with type “72” from the EM data set (Bae et al., 2018)^1^, which had 13.6 ± 1.2% (mean ± SD, *n* = 5) of its dendritic length between strata. In comparison, the ON delayed RGC (Figure 1B) and SbC RGC presented by Tien et al. (2015; Figure 1C) both stratify within the ON ChAT band and distal to the OFF ChAT band. The major distinguishing feature of these cells was their high degree of recursive dendrites accounting for a larger proportion of the total dendritic length between strata. OND RGCs had 39 ± 11% of their dendrites between strata, and the image provided to us from the Tien et al. (2015) publication had 38% of its dendrite length between strata. The stratification pattern of both the ON delayed RGC and the SbC RGC of Tien et al. (2015) matched type “73” from the EM data set including the fraction of dendrites between strata (33.5 ± 4.3%, mean ± SD, *n* = 6), and those authors confirmed the identify of type “73” as the ON delayed RGC (Bae et al., 2018).

## Function

After a RGC is assigned a response polarity, the most common secondary functional parameter used in classification is kinetics: whether the light response profile is transient or sustained. Response kinetics has been important in distinguishing the brisk-transient vs. brisk-sustained RGCs in the rabbit retina (Caldwell and Daw, 1978; Amthor et al., 1989a,b; Devries and Baylor, 1997), parasol vs. midget RGCs in the primate retina (Watanabe and Rodieck, 1989; Dacey, 1994), transient vs. sustained Alpha RGCs in the mouse retina (Pang et al., 2003; Murphy and Rieke, 2006), and the High Definition family (HD1, HD2, UHD) of ON-OFF RGCs in the mouse retina (Jacoby and Schwartz, 2017). We hypothesized that different types of SbC RGCs could also be distinguished based on the kinetics of their stimulus-dependent suppression.

**Figure 2:**
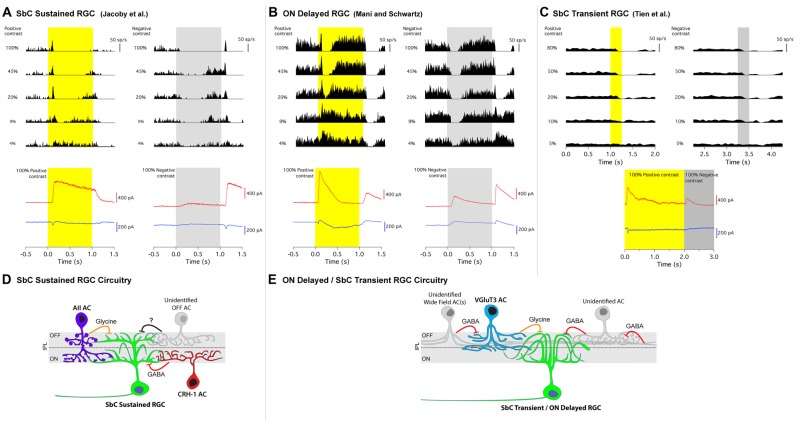
Contrast response profiles, intracellular currents and circuit diagrams of two types of SbC RGCs. **(A)** SbC sustained RGC, **(B)** ON delayed RGC, **(C)** SbC transient RGC. Top traces; cell attached spike responses to varying levels of Weber contrast stimuli to positive (left) and negative (right) contrast from mean illumination. Middle section; excitatory (blue) and inhibitory (red) intracellular currents in response to 100% positive and 100% negative contrast stimuli. Schematic circuit diagrams for the SbC sustained RGC **(D)** and ON delayed/SbC transient RGC **(E)**. Permission from the copyright holders was obtained for use and modification of previously published figures.

Jacoby et al. (2015) used a 1 s light step from darkness to classify SbC RGCs. The cells in this study were suppressed for the entire duration of this stimulus. Longer timescales were explored, and it was found that suppression for a light step from darkness could extend through the entire duration of a 20 s light step (Figure 1A, bottom). When these SbC RGCs were exposed to varying levels of positive/negative contrast spots from mean background illumination, suppression was sustained throughout the entire stimulus time for contrasts exceeding positive or negative 50% (Figure 2A).

SbC RGCs reported by Tien et al. (2015) suppressed their firing more transiently (Figure 2C). Suppression lasted roughly 0.5 s in duration to positive contrast stimuli that were presented for 2 s (Tien et al., 2015). This transient suppression profile of SbC transient RGCs closely resembles the “spike latency period” of the ON delayed RGC discovered by Mani and Schwartz (2017). In response to a 1 s light step from darkness, ON delayed RGCs were transiently suppressed for ~0.5 s (Figure 1B). For a 20 s light step firing in an ON delayed RGC resumed within 0.5 s (Figure 1B, bottom and inset zoom). In the ON delayed RGC, spike suppression was similarly transient for both positive and negative contrasts from a photopic background (Figure 2B).

Another group observed significant variability in the kinetics of contrast suppression in the population SbC RGCs they recorded in the mouse retina (Lee et al., 2016). In offering an explanation for this variability, they noted that “…temporal variability may be attributable to subtle differences in recording conditions and/or to an intrinsic cell-to-cell variability, but the existence of different functional subtypes of Uniformity Detector (SbC) RGCs also remains a possibility (Lee et al., 2016).” Indeed, multiple cell types may comprise the data presented in their article and account for the observed response variability; in Figure 3D of their publication the top four traces resemble the response profiles of ON delayed RGC or the transient SbC RGC identified by Tien et al. (2015), and the remainder resemble the more sustained SbC RGCs of Jacoby et al. (2015). Thus, interpretation of the pharmacology and circuit tracing results are complicated by unifying these two different cell types (see below).

To determine if the ON delayed RGC targeted in wild type retina target by Mani and Schwartz (2017) was the same cell type as the SbC transient RGC targeted in the CCK-cre transgenic line described by Tien et al. (2015), the authors of this review obtained the CCK transgenic mouse and recorded from fluorescently labeled RGCs. Despite several ganglion cell types being tagged with the fluorescent marker in this line, both morphological and physiological examination confirmed that ON delayed RGCs are indeed one of the RGC types labeled in CCK-cre mice (Figure 1D). The morphology and physiology of these cells recorded in the CCK-cre line were indistinguishable from ON delayed RGCs from wild type retina, but were distinct from the recordings of SbC sustained RGCs (Jacoby et al., 2015). Thus, we conclude that the cells reported by Tien et al. (2015) and in their subsequent article (Tien et al., 2016) are the same type as the ON delayed RGC reported by Mani and Schwartz (2017), and that the SbC sustained RGC type reported by Jacoby et al. (2015) is a different cell type altogether. We will subsequently refer to these cell types as “transient” and “sustained” SbC RGCs respectively, but we acknowledge that there may be additional SbC RGC types and that more precise nomenclature may be required to differentiate them in the future.

## Circuit Mechanisms of Contrast Suppression

All three groups that published on the SbC RGCs in mouse retina also explored the upstream circuit elements that contributed to the SbC computation. It is important to consider these results in the context of our contention that they stem from two different cell types that may or may not share circuit elements. By identifying specific presynaptic partners of SbC RGCs, these studies inform our understanding of the mechanisms of contrast suppression. We will review both the known and unknown elements of these pathways with emphasis on the mechanistic differences between the transient and sustained SbC circuits.

## Synaptic Inputs

To explore the excitatory and inhibitory synaptic inputs onto SbC RGCs, whole-cell voltage clamp was used to isolate these currents. A common characteristic of the synaptic currents in both SbC types was that inhibition was much larger than excitation (Figure 2). This was also true in previous reports of the rabbit Uniformity Detector RGC (Sivyer et al., 2010). Both mouse SbC RGC types had small excitatory currents at light onset, sometimes with separate transient and sustained components (Figure 2). Both cell types also showed a small, sustained decrease in tonic excitation at light offset. The lack of increased excitation is notable given that both SbC RGC types had a dendritic stratification in what is known as the OFF layer of the IPL. This constitutes a growing body of evidence that RGCs with dendrites in the outer half of the IPL do not necessarily receive input from OFF bipolar cells (Dumitrescu et al., 2009; Hoshi et al., 2009; Jacoby et al., 2015; Nath and Schwartz, 2016, 2017).

In the absence of inhibition, this pattern of excitation (increase for positive contrast and decrease for negative contrast) would yield an ON contrast response profile in the RGC’s spiking response, so inhibition must play a critical role in contrast suppression. Both SbC RGC types had a large inhibitory conductance at light onset that dominated the small excitatory conductance to yield a net hyperpolarization and decrease in spiking, but the ON inhibition differed in both kinetics and pharmacology between the two SbC RGC types. SbC sustained RGCs had a sustained inhibitory current that extended throughout the entirety of a 1 s light step (Figure 2A), while ON delayed and SbC transient RGCs had a transient inhibitory current at light onset that decayed to baseline within ~ 500 ms (Figures 2B,C), depending on the size of the light stimulus (Mani and Schwartz, 2017).

Inhibition at light onset in the SbC sustained RGC was driven by both GABA_A_ and/or GABA_C_ receptors (53%) and glycine receptors (47%; Jacoby et al., 2015). In the transient SbC RGC, the vast majority of inhibition at light onset was from glycine receptors (~75%) with only a very small GABAergic component remaining after glycine receptor block (~25%; Tien et al., 2015). However, these results are difficult to interpret because serial inhibition can cause non-additivity of glycine and GABA components (Figures 2D,E). Both SbC RGC types also received inhibition at light offset to support the small decrease in excitation in reducing spiking, but the OFF inhibition differed in relative amplitude between the two RGC types. The ratio of ON/OFF inhibition was 6.2 ± 1.4 in SbC sustained RGCs, vs. 2.5 ± 0.9 in SbC transient RGCs.

## Presynaptic Amacrine Cells

Not only were synaptic inputs identified, but specific presynaptic AC types were confirmed as sources of inhibition to help shape the contrast suppression profiles of SbC RGCs. Jacoby et al. (2015) identified that GABAergic CRH-1 ACs are direct presynaptic partners to the SbC sustained RGC through paired patch clamp recordings (Jacoby et al., 2015). The highly sustained nature of CRH-1 AC responses to light onset help to drive the sustained suppression of spike activity in SbC sustained RGCs throughout the duration of long visual stimuli (Figure 1A, bottom). When CRH-1 ACs surrounding a single SbC sustained RGC were physically ablated from circuit input, suppression to positive contrast was greatly reduced, and the SbC sustained RGC was converted into a stereotypical ON cell with its firing rate increasing to positive contrast (Jacoby et al., 2015). The authors also pharmacologically isolated AII ACs and showed that they supply some of the glycinergic input to sustained SbC RGCs at light onset. Conclusions from this study were that: (1) CRH-1 ACs are a necessary component of contrast suppression in SbC sustained RGCs; and (2) that AII ACs support suppression at light onset. The authors speculated that a different AC type or types provide the smaller inhibitory drive at light offset.

Both Tien et al. (2016) and Lee et al. (2016) confirmed that VGluT3 ACs release glycine onto transient SbC RGCs through optogenetics (both studies) and paired recordings. As noted above, Lee et al. (2016), likely combined both sustained and transient version of SbC RGC types in their study. When VGluT3 ACs were genetically ablated using diphtheria toxin, OFF (but not ON) inhibition was reduced in a size selective manner (for small but not large spots) and the time of spike suppression was reduced (Tien et al., 2016). The authors concluded that VGluT3 ACs play a role in contrast suppression at light offset for small stimuli in SbC transient RGCs, but that different ACs are involved in suppression at light onset and for large OFF stimuli. Schematics summarizing the inhibitory circuit elements identified upstream of both SbC RGC types are shown in (Figures 2D,E).

## Conclusion

SbC RGCs respond to increases and decreases in illumination by decreasing their baseline firing rate, and like the traditional ON, OFF, and ON-OFF response polarity classes, functional distinctions within the SbC class depend on characteristics like response kinetics. Along with the rapid identification of these two SbC cell types in mouse retina, the three groups working on these cells also revealed some of the ACs responsible for the SbC computation.

We provided evidence that this SbC cell class is comprised of at least two distinct cell types. A highly-sustained SbC RGC type was identified in wild type mice by Jacoby et al. (2015) and has distinct morphological, functional, synaptic inputs and circuit connectivity when compared to the SbC transient RGC identified by Tien et al. (2015) and the ON delayed RGC identified by Mani and Schwartz (2017). We show several lines of evidence that the ON delayed and SbC transient RGCs are the same cell type.

It is possible that other types of SbC RGCs exist in the mouse retina. Just as has been shown in the ON, OFF, and ON-OFF polarity classes, SbC RGCs may each fill a specific niche in kinetics and perhaps other parameters such as stimulus size, motion speed and color.

## Author Contributions

JJ and GS conceived this manuscript idea, wrote the manuscript and created the figures.

## Conflict of Interest Statement

The authors declare that the research was conducted in the absence of any commercial or financial relationships that could be construed as a potential conflict of interest.

## Footnotes

^1^http://museum.eyewire.org
